# Validation of a description of sarcopenic obesity defined as excess adiposity and low lean mass relative to adiposity

**DOI:** 10.1002/jcsm.12613

**Published:** 2020-09-15

**Authors:** Joshua F. Baker, Tamara Harris, Allegra Rapoport, Susan L. Ziolkowski, Mary B. Leonard, Jin Long, Babette Zemel, David R. Weber

**Affiliations:** ^1^ Division of Rheumatology Philadelphia Veterans' Affairs Medical Center Philadelphia PA USA; ^2^ Division of Rheumatology, School of Medicine University of Pennsylvania 8 Penn Tower Building, Philadelphia PA USA; ^3^ Center for Clinical Epidemiology and Biostatistics University of Pennsylvania Philadelphia PA USA; ^4^ Laboratory of Epidemiology and Population Sciences, Intramural Research Program NIA, NIH Bethesda MD USA; ^5^ John's Hopkins School of Public Health Baltimore MA USA; ^6^ Department of Medicine and Pediatrics Stanford University Palo Alto CA USA; ^7^ Children's Hospital of Philadelphia Philadelphia PA USA; ^8^ Division of Endocrinology and Diabetes Golisano Children's Hospital, University of Rochester Rochester NY USA

**Keywords:** Sarcopenia, Obesity, Physical function

## Abstract

**Background:**

This study aims to assess the construct validity of a body composition‐defined definition of sarcopenic obesity based on low appendicular lean mass relative to fat mass (ALMI_FMI_) and high fat mass index (FMI) and to compare with an alternative definition using appendicular lean mass index (ALMI) and percent body fat (%BF).

**Methods:**

This is a secondary analysis of two cohort studies: the National Health and Examination Survey (NHANES) and the Health, Aging, and Body Composition study (Health ABC). Sarcopenic obesity was defined as low ALMI_FMI_ combined with high FMI and was compared with a widely used definition based on ALMI and %BF cut‐points. Body composition *Z*‐scores, self‐reported disability, physical functioning, and incident disability were compared across body composition categories using linear and logistic regression and Cox proportional hazards models.

**Results:**

Among 14, 850 participants from NHANES, patients with sarcopenic obesity defined by low ALMI_FMI_ and high FMI (ALMI_FMI_‐FMI) had above‐average FMI *Z*‐scores [mean (standard deviation): 1.00 (0.72)]. In contrast, those with sarcopenic obesity based on low ALMI and high %BF (ALMI‐%BF) had below‐average FMI *Z*‐scores. A similar pattern was observed for 2846 participants from Health ABC. Participants with sarcopenic obesity based on ALMI_FMI_‐FMI had a greater number of disabilities, worse physical function, and a greater risk of incident disability compared with those defined based on ALMI‐%BF.

**Conclusions:**

Body composition‐defined measures of sarcopenic obesity defined as excess adiposity and lower‐than‐expected ALMI relative to FMI are associated with functional deficits and incident disability and overcome the limitations of using %BF in estimating obesity in this context.

## Introduction

Sarcopenic obesity is a frequently used term that aims to describe individuals with both evidence of muscle loss and excess adiposity. Sarcopenic obesity is thought to occur in the setting of aging and chronic illness^1^, ^2^, ^3^ and is often considered to be a distinct phenotypic presentation from low muscle mass (i.e. sarcopenia) alone.

A number of body composition‐derived definitions of sarcopenic obesity have been developed, resulting in wide variability in the prevalence of this condition.^4^, ^5^, ^6^, ^7^, ^8^, ^9^ Many studies evaluating sarcopenic obesity identify obese individuals on the basis of percent body fat (%BF).^4^, ^7^ However, this criterion for categorizing obesity is confounded by the presence of sarcopenia. Among individuals with the same fat mass, %BF will be higher in those with lower muscle mass (%BF = fat mass/(fat mass + fat‐free mass)). Therefore, this criterion is insensitive to the fact that severe reductions in muscle mass alone will result in a high %BF. This has the potential to result in the misclassification of many of these individuals as obese. The use of fat mass index (FMI, kg/m^2^) to define obesity overcomes this limitation.

However, a strong positive and non‐linear correlation between muscle and fat exists such that obese individuals are expected to have both higher fat mass and muscle mass compared with non‐obese individuals.^10^ Therefore, the combination of very low lean mass with excess fat mass is very uncommon. We recently developed a novel measure of appendicular lean mass relative to fat mass (ALMI_FMI_) that represents the deficit in muscle relative to an individual's level of adiposity and overcomes these confounding associations between lean and fat.^10^, ^11^, ^12^ This novel construct builds on prior approaches by Newman *et al*.^13^ and others and correlates strongly with physical function and incident disability in older people and in rheumatoid arthritis independently of measures of fat mass.^11^, ^12^ The strengths of this approach also include consideration of non‐linear relationships and differences in relationships between lean and fat between sex and racial groups. The development of this validated definition of relative sarcopenia provides a new opportunity to define sarcopenic obesity as the combination of obesity and relative deficits in lean mass. We therefore propose a body composition‐derived definition of sarcopenic obesity that represents the presence of obesity (defined by FMI) and a lower‐than‐expected muscle mass (low ALMI_FMI_) based on these prior validated methods.

We aimed to compare this novel definition of sarcopenic obesity with other previously used definitions based on %BF and to describe body composition parameters using each definition using two well‐characterized datasets. We also assessed the strength of association of each definition of sarcopenic obesity with physical functioning and incident disability. We hypothesized that the novel definition of sarcopenic obesity would strongly correlate with poor physical function and incident disability.

## Methods

### National Health and Nutrition Examination Survey body composition cohort

We analysed data from 14 850 National Health and Nutrition Examination Survey (NHANES) participants aged 20 to 85 with dual energy X‐ray absorptiometry (DXA) body composition data obtained between 1999 and 2006. NHANES includes a nationally representative sample of the US population and is designed to determine the health and nutritional status of people of all ages. The survey consists of questionnaires administered in the home and standardized physical health examinations in specially equipped mobile examination centres. The National Center for Health Statistics (NCHS) Research Ethic Review Board reviews and approves the NHANES protocol annually.

### Health aging and body composition study cohort

In order to test the performance of the constructs in another cohort at risk of sarcopenia, we utilized data from the Health, Aging and Body Composition (Health ABC) study: a prospective observational study of 3075 well‐functioning, community‐dwelling older adults aged 70–79 years. Study participants were recruited from a random sample of White and all Black Medicare beneficiaries living in selected zip codes in Pittsburgh, PA, and Memphis, TN. Participants were eligible for enrolment if they self‐reported no difficulty walking a quarter‐mile, climbing 10 steps, or performing activities of daily living. Persons who reported a life‐threatening illness, were undergoing active cancer treatment, required an assistive walking device, or had plans to leave the geographic area within 3 years were excluded. All individuals with available body composition data at baseline were included in this analysis (*N* = 2846).

### Whole‐body dual energy X‐ray absorptiometry assessment of body composition (National Health and Nutrition Examination Survey)

Whole‐body DXA scans were acquired using Hologic QDR 4500A fan‐beam densitometers (Hologic, Inc., Bedford, MA). DXA exclusion criteria included pregnancy, weight >300 pounds (136 kg, due to the weight limit of the scanner), height >77 inches (195 cm), recent nuclear medicine scan, or exposure to radioactive contrast. Whole‐body quality control phantoms were scanned at least weekly. Each participant and phantom scan were reviewed and analysed by the NHANES quality control centre at the University of California San Francisco (UCSF) using standard radiologic techniques and study‐specific protocols. DXA scans were not obtained in patients with non‐removable objects (prostheses, pacemakers, implants, and casts), and some scans were excluded due to technical errors such as suboptimal participant positioning. To account for potential biases of non‐random missing data, multiple imputation was performed by the NCHS for all participants with invalid or missing data (with the exception of pregnant women) using demographic, socioeconomic, and geographic variables, body measurements, health indicators, dietary and medication use variables, and blood test results.^14^, ^15^

Prior studies demonstrated that the original algorithm for body composition determination underestimated fat mass and overestimated lean mass compared with criteria measurements of body composition from total body water by dilution, underwater weighing, and four‐compartment analysis.^16^ As is standard practice based on these prior data, the NHANES DXA lean mass was therefore decreased by 5%, and an equivalent kilogram weight was added to the fat mass, so total mass did not change.

The DXA measures of body composition included appendicular lean mass index (ALMI, kg/m^2^) and whole‐body fat mass index (FMI, kg/m^2^) as well as %BF.

### Whole‐body dual energy X‐ray absorptiometry assessment of body composition (Health ABC)

Dual energy X‐ray absorptiometry was performed at both Pittsburgh and Memphis field centres (Hologic 4500A, version 9.03; Hologic, Inc., Waltham, MA). Bone mineral‐free ALMI and total FMI were derived from the whole‐body scan. DXA quality assurance measurements were performed at both study sites to ensure scanner reliability, and identical patient scan protocols were used for all participants. A quality control centre was at the University of California, San Francisco. A total body phantom was exchanged between sites, and several volunteers were scanned periodically at both sites. For soft tissue, the coefficients of variation were 1.0% and 2.1% for whole‐body lean mass and fat mass, respectively.

### Generation of appendicular lean mass index, fat mass index, and appendicular lean mass relative to fat mass standard deviation scores

Sex and race/ethnicity‐specific LMS curves for ALMI and FMI relative to age were previously published by investigators at Hologic, Inc. using NHANES data.^17^, ^18^, ^19^ These curves were used to convert the ALMI and FMI results in this study to sex and race/ethnicity‐specific *Z*‐scores relative to age and to *T*‐scores based on LMS values in a 25‐year‐old.

Appendicular lean mass relative to fat mass *Z*‐scores and *T*‐scores were generated using previously published equations defined within NHANES. These equations adjust the ALMI *Z*‐scores and *T*‐scores for FMI *Z*‐ and *T*‐scores, respectively.^10^ The ALMI_FMI_ *Z*‐scores incorporate the complex interactions among age, sex, and race/ethnicity and represent the number of standard deviations (SDs) above or below the mean for a reference group of the same age, sex, race/ethnicity, and FMI *Z*‐score. The ALMI_FMI_ *T*‐scores conceptually represent the number of SDs above or below the mean for individuals 25 years of age of the same sex, race/ethnicity, and FMI *T*‐score.

### Definitions of low lean mass

We defined low ALMI_FMI_ based on prior work.^10^, ^12^ This prior work demonstrated that a low ALMI_FMI_ was associated with lower physical performance scores and a higher risk of incident disability among older adults as well as greater disability among patients with rheumatoid arthritis. Low lean *for age* relative to adiposity was defined as a ALMI_FMI_ *Z*‐score of ≤−1 (15.9th percentile for the NHANES population). Low lean relative to adiposity was also alternatively defined as an ALMI_FMI_ *T*‐score of ≤−2. For comparative purposes, we also defined sarcopenia using the commonly cited Baumgartner definition, ALMI (kg/m^2^) more than two SDs below the mean in a young reference population (18–40 years of age) from the Aging Process Study and the Rosetta Study (men: 7.26 kg/m^2^, women: 5.45 kg/m^2^).^20^

### Definitions of obesity

Obesity was also defined based on sex‐specific FMI cut‐point values developed by Kelly *et al*. in order to generate a prevalence of obesity that was the same as observed using a BMI cut‐point of 30 kg/m^2^ in 25‐year‐old participants in NHANES (FMI ≥13 for women; ≥9 for men).^17^ These cutoffs performed similarly across racial groups. Based on previous publications, we also used the definition of obesity based on %BF (≥40% for women; ≥30% for men) (The American Council on Exercise).^21^, ^22^

### Definitions of sarcopenic obesity

We used a novel method of categorizing body composition based on ALMI_FMI_ and FMI (termed ALMI_FMI_‐FMI). We compared this new method with a commonly used method using a combination of ALMI and %BF (termed ALMI‐%BF). For both approaches, sarcopenic obesity was defined as the presence of both low lean mass and obesity (see respective definitions earlier).

### Assessment of physical functioning in National Health and Nutrition Examination Survey

Questions about physical functioning were asked as a part of a home interview in a subset of individuals between 2001 and 2014. Participants are asked to report how much difficulty they experience performing daily activities important for functional independence. Similar to prior studies,^23^ we incorporated six questions from this questionnaire felt to be examples of difficulties likely to be encountered in individuals with adverse body composition including (i) climbing 10 steps; (ii) walking a quarter‐mile; (iii) stooping, crouching, or kneeling; (iv) standing from an armless chair; (v) lifting or carrying heavy objects; and (vi) standing for long periods of time. We considered a physical functioning limitation to be present if the patient reported any difficulty with these activities. The number of questions for which the participant reported difficulty were summed and categorized as 0, 1–2, 3–4, or 5–6.

### Physical function and incident disability in Health ABC

The Health ABC performance battery includes five repeated chair stands, progressively more challenging tests of standing balance, a 6 m walk to determine usual gait speed, and a narrow walk in which participants are instructed to walk between lines of coloured tape 20 cm apart at their usual pace.^24^, ^25^ Performance is divided by the maximum possible performance for older adults on each test to create ratio scores that are summed for the four tests to obtain a continuous scale ranging from 0 to 4, with a lower score indicating poorer function. The minimally important difference in the Health ABC Score is estimated to be 0.15 based on previous studies of the Short Physical Performance Battery.^26^

Adjudicated self‐report data on incident physical disability were obtained from interviewer‐administered questionnaires every 6 months.^27^, ^28^ For physical disability, the outcome of interest was time from baseline (Visit 1) to any self‐reported disability, which was defined as severe difficulty or inability to walk 1/4 mile and/or climb 10 steps, needing equipment to ambulate, or having any difficulty performing activities of daily living (i.e. getting in and out of bed or chairs, bathing or showering, and dressing).

### Statistical analysis

Body composition *Z*‐scores (ALMI, ALMI_FMI_, and FMI) and per cent fat were described over the four categories of both composition based on each classification method (either ALMI_FMI_‐FMI or ALMI‐%BF). The four categories of body composition included (i) normal body composition, (ii) low lean mass only, (iii) obesity only, and (iv) sarcopenic obesity (both present). In addition, the proportion of patients reporting difficulty with specific tasks related to physical functioning from NHANES was described over each body composition category. Proportions were compared using *χ* ^2^ tests and logistic regression. Ordinal regression compared the odds of reporting difficulty with a greater number of tasks (categorized as 0, 1–2, 3–4, or 5–6) adjusting for age, sex, and race.

In Health ABC, body composition *Z*‐scores were similarly compared across categories. The Health ABC performance score and the subsequent risk of incident disability were also assessed in each category compared with normal using linear regression and Cox proportional hazards models, respectively, adjusting for age, sex, and race.

We also evaluated the performance of categorization methods based on ALMI_FMI_ *T*‐scores in similar analyses (presented in the supporting information). Analyses were carried out using Stata 14.2 software (StataCorp, LP, College Station, TX). Analyses included imputed body composition data and were performed using sample weights to account for the complex sample design as recommended by the NCHS.^29^

## Results

### Prevalence of sarcopenic obesity by different definitions in National Health and Nutrition Examination Survey

The categorization of body composition using different strategies is shown in *Table* 1. As expected, low ALMI and high FMI occurred together rarely; only 0.65% of the population had both (not shown in the table). The ALMI‐%BF categorization approach resulted in 1404 (9%) participants categorized as low lean mass only and 543 (4%) categorized as sarcopenic obesity in the population. The ALMI_FMI_‐FMI approach resulted in 1495 (10%) participants being categorized as low lean mass and 621 (4%) categorized as sarcopenic obesity. There was moderate agreement between the two methods for categorizing body composition (*Table* 2) (kappa = 0.51). Notably, 2204 participants classified as either obese or low lean based on the ALMI‐%BF method were considered normal by the ALMI_FMI_‐FMI method. In addition, 594 patients identified as obese only based on the ALMI‐%BF method were considered to have sarcopenic obesity based on the ALMI_FMI_‐FMI method.

**Table 1:** The number and per cent of participants categorized as low lean, obese, or sarcopenic obese by each method and the body composition *Z*‐scores and per cent body fat across these categories (mean, standard deviation) for each categorization method in National Health and Nutrition Examination Survey

	*N* (%)	ALMI *Z*‐score	ALMI_FMI_ *Z*‐score	FMI *Z*‐score	% fat
ALMI and %BF^a^	14 823				
Normal	6448 (44%)	−0.083 (1.06)	0.44 (1.35)	−0.52 (1.12)	29 (7.41)
Low lean only	1404 (9%)	−1.58 (0.66)	−1.10 (1.12)	−1.29 (0.77)	28 (8.23)
Obese only	6428 (43%)	0.55 (1.44)	−0.058 (1.22)	0.82 (1.08)	41 (7.32)
Sarcopenic obesity	543 (4%)	−1.41 (0.73)	−1.81 (1.26)	−0.10 (0.41)	39 (6.11)
ALMI_FMI_ *Z*‐score and FMI^b^	14 850				
Normal	8301 (56%)	−0.20 (1.01)	0.28 (1.35)	−0.52 (1.10)	30 (9.96)
Low lean only	1495 (10%)	−1.57 (0.72)	−1.64 (0.90)	−0.61 (0.82)	33 (9.43)
Obese only	4433 (30%)	0.96 (1.15)	0.24 (1.03)	1.06 (0.85)	41 (9.02)
Sarcopenic obesity	621 (4%)	−0.37 (0.73)	−1.56 (0.73)	1.00 (0.72)	42 (7.10)

^a^ Low ALMI defined as <7.26 kg/m^2^ for men and <5.45 kg/m^2^ for women.^20^ High %BF defined as ≥30% for men, ≥40% for women.^30^

^b^ Low ALMI_FMI_ *Z*‐score defined as an ALMI_FMI_ *Z*‐score ≤−1.^10^ High FMI defined as ≥9 kg/m^2^ for men, ≥13 kg/m^2^ for women.^17^

**Table 2:** Agreement between traditional methods of categorizing body composition with the novel categorization method in National Health and Nutrition Examination Survey

		ALMI_FMI_‐FMI				
	Agreement = 73.5%, Kappa = 0.58	Normal	Low lean only	Obese only	Sarcopenic obesity	Total
ALMI‐%BF	Normal	5705	177	213	0	6095
	Low lean only	711	667	0	0	1378
	Obese only	1493	250	4406	554	6703
	Sarcopenic obesity	132	381	17	118	648
	Total	8041	1475	4636	672	14 824

### Body composition by different definitions of sarcopenic obesity in National Health and Nutrition Examination Survey

**Figure 1:**
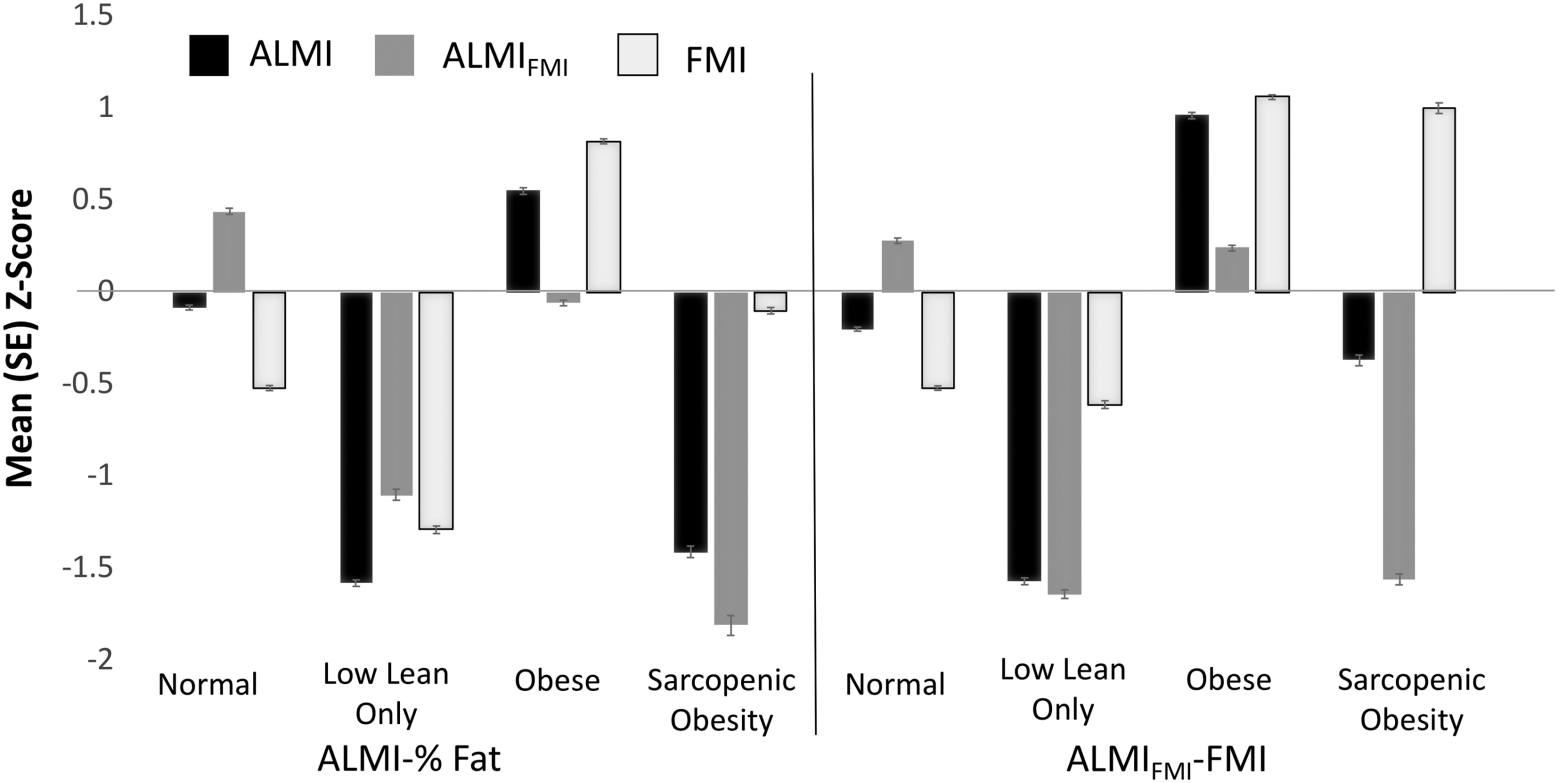
Body composition among participants from National Health and Examination Survey categorized by ALMI‐%BF and ALMI_FMI_‐FMI methods.

Participants with sarcopenic obesity defined by either the ALMI‐%BF or ALMI_FMI_‐FMI method had low ALMI and ALMI_FMI_ *Z*‐scores (*Table* 1, *Figure* 1). However, fat mass was below average (*Z*‐score <0) in individuals with sarcopenic obesity defined by ALMI‐%BF [FMI *Z*‐score: −0.10 (0.41)] and was above average for the population with sarcopenic obesity defined by ALMI_FMI_‐FMI [FMI *Z*‐score: 1.00 (0.72)] (*P* < 0.0001). Similar results were observed when using *T*‐scores to categorize and describe body composition (Supporting Information, *Tables* *S1* and *S2*).

### Definitions of sarcopenic obesity and associations with reported function in National Health and Nutrition Examination Survey

For some activities, participants with sarcopenic obesity by the ALMI‐%BF definition reported higher disability compared with those with low lean mass alone. However, they were often less likely to report disability compared with those with obesity alone. In contrast, sarcopenic obesity based on the ALMI_FMI_‐FMI was generally associated with numerically greater disability compared with low lean mass alone or obesity alone (Supporting Information, *Tables* *S3* and *S4*).

**Figure 2:**
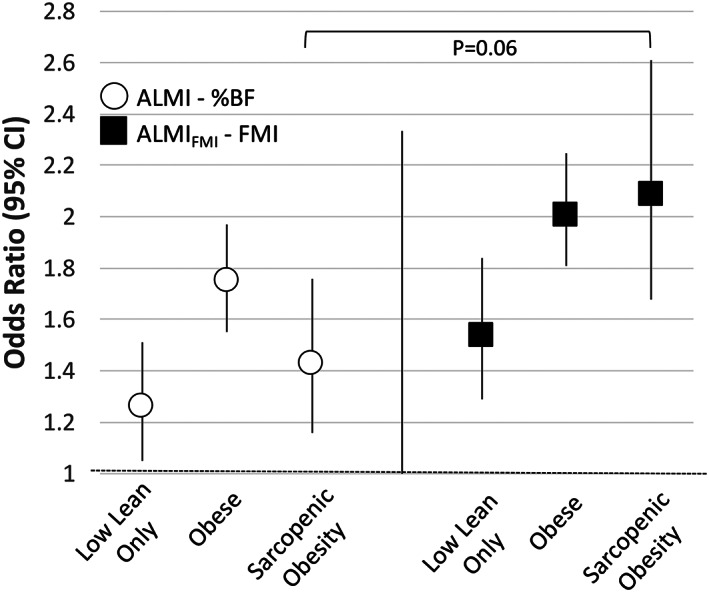
The risk of a greater number of self‐reported disabilities by body composition category (compared with normal as reference) using ordinal regression among participants in National Health and Examination Survey.

In ordinal regression models, participants categorized as low lean mass, obese, or sarcopenic obesity by the ALMI_FMI_‐FMI method all had a higher risk of disability compared with normal body composition. Individuals with sarcopenic obesity based on ALMI_FMI_‐FMI had the greatest likelihood of reporting greater disability [odds ratio: 2.09 (1.68, 2.59) *P* < 0.001] (*Figure* 2). For the ALMI‐%BF method, those with sarcopenic obesity had a more modest increase in risk of disability compared with normal body composition [odds ratio: 1.45 (1.18, 1.78) *P* < 0.001] (*P* = 0.06 for the comparison with the regression coefficient for ALMI_FMI_‐FMI). In addition, the risk of sarcopenic obesity based on ALMI‐%BF was lower than that for obesity alone (*P* = 0.04). Results using a categorization method using ALMI_FMI_ *T*‐scores are shown in Supporting Information, *Table* S5.

### Validation in Health ABC

The frequency of sarcopenic obesity and the body composition patterns observed in the Health ABC study were similar to those observed in NHANES (*Table* 3). For example, approximately 3% of the population was defined as having sarcopenic obesity using both the ALMI‐%BF and ALMI_FMI_‐FMI definitions. Obesity was much more prevalent when defined based on %BF as opposed to FMI (49% vs. 29%).

**Table 3:** Body composition *Z*‐scores across body composition categories for each definition in Health ABC

	*N* (%)	ALMI *Z*‐score	ALMI_FMI_ *Z*‐score	FMI *Z*‐score	% fat
ALMI and % fat	2844				
Normal	1249 (44%)	−0.049 (0.73)	0.73 (0.88)	−0.84 (0.58)	30.2(5.83)
Low lean only	192 (7%)	−1.54 (0.53)	−0.94 (0.76)	−1.53 (0.59)	28.4 (6.17)
Obese only	1317 (46%)	0.26 (0.77)	0.02 (0.87)	0.44 (0.62)	40.1 (6.12)
Sarco obese	86 (3%)	−1.48 (0.52)	−1.76 (0.72)	−0.24 (0.52)	37.7 (5.40)
ALMI_FMI_ *Z*‐score and FMI	2844				
Normal	1810 (64%)	−0.16 (0.67)	0.46 (0.85)	−0.70 (0.65)	32.2 (6.97)
Low lean only	213 (7%)	−1.65 (0.56)	−1.67 (0.60)	−0.81 (0.66)	36.7 (6.54)
Obese only	716 (25%)	0.76 (0.64)	0.40 (0.81)	0.80 (0.48)	39.9 (6.49)
Sarco obese	105 (4%)	−0.48 (0.63)	−1.52 (0.56)	0.87 (0.51)	44.2 (6.28)

Participants with sarcopenic obesity based on ALMI‐%BF had very low ALMI and ALMI_FMI_ *Z*‐scores but had FMI *Z*‐scores that were below average compared with national averages [FMI *Z*‐score: −0.24 (0.52)] (*Table* 3). Participants with sarcopenic obesity based on ALMI_FMI_‐FMI had low ALMI *Z*‐scores, very low ALMI_FMI_ *Z*‐scores, and FMI *Z*‐scores that were much above average [FMI *Z*‐score: 0.87 (0.51)].

Participants with sarcopenic obesity based on ALMI_FMI_‐FMI had inferior physical performance adjusting for age, sex, and race (*Table* 4). Performance scores were numerically lower among those with sarcopenic obesity based on ALMI_FMI_‐FMI [mean (95% confidence interval): 1.91 (1.82, 2.01)] when compared with those with sarcopenic obesity based on ALMI‐%BF [mean (95% confidence interval): 2.00 (1.90, 2.11)] (*P* for comparison 0.32). Sarcopenic obesity based on ALMI_FMI_‐FMI was also associated with the highest risk of incident disability for all groups studied after adjusting for age, sex, and race [hazard ratio 1.70 (1.37, 2.11) *P* < 0.001] (*Table* 4). This was not statistically different than the risk observed for the sarcopenic obesity defined by ALMI‐%BF [hazard ratio 1.32 (1.03, 1.70)] (*P* for comparison = 0.07).

**Table 4:** Linear regression and Cox proportional hazards model evaluating risks of poor physical function and incident disability

	Health ABC performance score	Risk of incident disability (*N* = 2843, 2097 events)
	Predicted mean (95% CI)	HR (95% CI)
ALMI and % fat		
Normal	2.30 (2.27, 2.33)	1 (reference)
Low lean only	2.21 (2.14, 2.28)^*^	1.18 (0.98, 1.42)
Obese only	2.12 (2.10, 2.15)^***^	1.44 (1.32, 1.58)^***^
Sarcopenic obesity	2.00 (1.90, 2.11)^***^	1.32 (1.03, 1.70)^*^
ALMI_FMI_ *Z*‐score and FMI		
Normal	2.28 (2.25, 2.30)	1 (reference)
Low lean only	2.13 (2.06, 2.19)^***^	1.24 (1.06, 1.46)^**^
Obese only	2.09 (2.05, 2.12)^***^	1.53 (1.38, 1.69)^***^
Sarcopenic obesity	1.91 (1.82, 2.01)^***^	1.70 (1.37, 2.11)^***^

Table shows predicted mean of the Health ABC physical function score from regression models in each group and HR for incident disability adjusting for age, sex, and race.

* p<0.05;

** p<0.01;

*** p<0.001.

## Discussion

This study supports the use of a body composition‐derived definition of sarcopenic obesity represented as a deficit in lean mass relative to fat mass in an individual with excess fat mass. This study found that, while the prevalence of participants with sarcopenic obesity was similar with the different approaches, our novel definition that used FMI to define obesity correctly identified individuals with above‐average adiposity. In contrast, a definition of obesity based on %BF identified individuals with below‐average adiposity. Furthermore, the novel definition was associated with functional deficits, including poor reported function, poor physical performance, and incident disability. The novel definition is conceptually distinct from prior proposed categorization methods in that it defines sarcopenic obesity as excess adiposity and a *lower‐than‐expected* lean mass for the level of adiposity.

Misclassification can occur when defining obesity based on per cent fat among individuals who have low lean mass (as is commonly done) because reductions in lean mass will result in increased %BF even when total fat mass is normal or low. Thus, patients with the most severe reductions in lean mass will also have a higher likelihood of having a high %BF. The current study illustrates that definitions of sarcopenic obesity that utilize %BF identify a group of people that have, on average, normal adiposity for their age. A complication of using absolute measures of total lean and fat, however, is that very few patients who have low absolute lean mass will also have excess total fat mass due to strong positive relationships between lean and fat. This is illustrated in the current study where <1% of individuals had both low ALMI and high FMI by previous DXA definitions. The novel definition presented here overcomes this longstanding challenge by utilizing a recently validated construct of low lean mass *relative* to fat mass. As noted, this approach conceptually defines sarcopenic obesity as high fat mass in combination with a low lean mass relative to fat mass.

Newman *et al*. initially proposed a definition of sarcopenia that was defined based on the residuals of lean mass to fat mass.^13^, ^31^ The method utilized in the current study to define sarcopenia builds on this work because it more clearly defines sex‐specific and race‐specific relationships and non‐linear relationships between lean and fat in the NHANES reference population (a nationally representative sample).^10^, ^11^ This method has been validated in older individuals and in patients with arthritis.^11^, ^12^ The current study is a further advance by supporting the use of a construct of sarcopenic obesity based on body composition measures that is operationalized as the combination of relative sarcopenia with previously validated definitions of excess fat mass. In this study, low lean mass, obesity, and sarcopenic obesity as defined by the novel approach more clearly identified participants who had functional impairment or who were at risk for functional impairment.

The novel construct identified approximately 5% of the national population as having sarcopenic obesity. Thus, the approach identifies a significant portion of the population that may benefit from public health interventions addressing these particular adverse body composition changes. An important area of future study will be to determine how targeted approaches for managing sarcopenia and obesity in the population can be employed to address the potentially unique characteristics of this population that suffers from both.

An important limitation of the current study is the lack of consideration of visceral adiposity. It has been shown that adverse changes in fat distribution may be observed in patients with sarcopenia and that these changes can contribute to functional impairment.^32^, ^33^ Our novel definition of sarcopenic obesity using ALMI_FMI_‐FMI, as with prior definitions, does not consider the distribution of fat. An additional limitation of the proposed method is that it currently requires some computational work once body composition measures are obtained. However, this computation could be very easily automated and incorporated in DXA software in the future. Finally, while the goal of this study was to assess the performance of this novel approach compared with an accepted alternative, the current study did not compare the novel construct to all other body composition‐defined descriptions of sarcopenic obesity. Further, while the study suggests that the novel construct identifies limitations in physical function and predicts incident disability at least as well as an existing construct, not all direct comparisons suggested statistical superiority, and the importance of these differences when translated to clinical practice and other population‐based cohorts remains unclear.

Another source of inconsistency in the definition of sarcopenic obesity in the current literature stems from a lack of a gold standard and the use of both imaging (densitometry) and functional definitions. The definition proposed here is one that is based on body composition characteristics alone. However, clinical definitions of sarcopenic obesity are likely to benefit from criteria that include assessments of strength or function. We suggest that the proposed categorization method might be combined with functional criteria to identify individuals adversely effected by sarcopenia and obesity. How body composition estimates and functional measures can be combined to more completely define the construct of sarcopenic obesity is an important question for future study. In addition, how to incorporate these criteria into distinct populations and diseases remains unclear and represents an important area of future study.^34^

In conclusion, sarcopenic obesity can be defined as excess adiposity in the setting of lower‐than‐expected lean mass. This definition correlates strongly with physical function and incident disability suggesting it represents a relevant and important construct. Definitions of sarcopenic obesity that incorporate measures of %BF are likely to misclassify individuals with low muscle mass as obese and show poorer correlation with physical functioning.

## Conflicts of interest

The authors have no conflicts to disclose.

## Funding

This research was supported by National Institute on Aging (NIA) Contracts N01‐AG‐6‐2101, N01‐AG‐6‐2103, and N01‐AG‐6‐2106; NIA grant R01‐AG028050; and National Institute of Nursing Research grant R01‐NR012459. This research was funded in part by the Intramural Research Program of the NIH, National Institute on Aging. Dr Baker has been supported by a Veterans Affairs Clinical Science Research and Development Career Development Award and Merit Award (IK2 CX000955 and I01 CX001703). Dr Leonard is supported by a K24 award (K24 DK076808). Dr Weber is supported by a K23 award (K23 DK114477). Dr Ziolkowski is supported by a F32 (F32 DK111083‐02).

## Supporting information


**Table S1.** Body composition T‐Scores across body composition categories defined based on ALMI and %BF categorization method in NHANES and Health ABC. For NHANES, the predicted means are presented with 95% CI.
**Table S2.** Body composition T‐Scores across body composition categories defined based on ALMI_FMI_ and FMI categorization method in NHANES and Health ABC.
**Table S3.** Percent of participants in NHANES with difficulty completing daily life tasks across body composition categories for each definition.
**Table S4.** Percent of participants from NHANES with difficulty completing daily life tasks across body composition categories. Analysis adjusted for sex and race only (T‐Score analysis).
**Table S5.** Ordinal regression model evaluating odds of greater disability among different categories of body composition by different methods adjusting for age, sex, and race (T‐Score analysis from NHANES).
**Table S6.** Linear regression and Cox proportional hazards model evaluating risks of poor physical function and incident disability adjusted for age, sex, and race. Table shows predicted mean of the Health ABC physical function score from regression models in each group and HR for incident disability.
