# Supplementary material for: Validation of a description of sarcopenic obesity defined as excess adiposity and low lean mass relative to adiposity
Source: J Cachexia Sarcopenia Muscle. 2020 Sep 15;11(6):1580–9. doi: 10.1002/jcsm.12613 (PMC7749601; doi:10.1002/jcsm.12613)
Supplement: Supplementary file 1 — Table S1. Body composition T‐Scores across body composition categories defined based on ALMI and %BF categorization method in NHANES and Health ABC. For NHANES, the predicted means are presented with 95% CI. Table S2. Body composition T‐Scores across body composition categories defined based on ALMIFMI and FMI categorization method in NHANES and Health ABC. Table S3. Percent of participants in NHANES with difficulty completing daily life tasks across body composition categories for each definition. Table S4. Percent of participants from NHANES with difficulty completing daily life tasks across body composition categories. Analysis adjusted for sex and race only (T‐Score analysis). Table S5. Ordinal regression model evaluating odds of greater disability among different categories of body composition by different methods adjusting for age, sex, and race (T‐Score analysis from NHANES). Table S6. Linear regression and Cox proportional hazards model evaluating risks of poor physical function and incident disability adjusted for age, sex, and race. Table shows predicted mean of the Health ABC physical function score from regression models in each group and HR for incident disability. [file JCSM-11-1580-s001.docx]

**Supplementary material**

Supplementary Table 1: Percent of participants in NHANES with difficulty completing daily life tasks across body composition categories for each definition.

|  | **Climb 10 Steps** | **Stoop Down** | **Lift Objects** | **Stand from Chair** | **Stand for Long Periods** | **Walk a ¼ Mile** |
| --- | --- | --- | --- | --- | --- | --- |
| **Low ALMI & High % Fat** | |  |  |  |  |  |
| Normal | 14.1 | 41.8 | 23.3 | 17.7 | 35.9 | 20.1 |
| Low Lean Only | 22.1*** | 45.5 | 33.1*** | 22.9** | 41.7** | 25.6** |
| Obese Only | 25.9*** | 58.9*** | 28.5*** | 30.3*** | 48.1*** | 32.2*** |
| Sarcopenic Obesity | 21.2** | 51.8*** | 32.9*** | 27.3*** | 47.1*** | 27.0** |
| **Low ALMI_FMI_ Z-Score & High FMI** | |  |  |  |  |  |
| Normal | 15.4 | 42.6 | 24.2 | 18.4 | 36.5 | 20.4 |
| Low Lean Only | 23.2*** | 53.4*** | 34.5*** | 28.2*** | 48.3*** | 28.2*** |
| Obese Only | 29.0*** | 62.8*** | 29.7*** | 33.1*** | 51.0*** | 35.8*** |
| Sarcopenic Obesity | 30.6*** | 62.0*** | 35.8*** | 35.5*** | 54.0*** | 38.9*** |

Supplementary Table 2: Body composition T-Scores across body composition categories defined based on ALMI and %BF categorization method in NHANES and Health ABC. For NHANES, the predicted means are presented with 95% CI.

|  | **N (%)** | **ALMI T** | **ALMI_FMI_ T** | **FMI T** |
| --- | --- | --- | --- | --- |
| **NHANES** | 14823 |  |  |  |
| Normal | 6448 (44%) | -0.082 (-0.11, -0.056) | 0.21 (0.17, 0.24) | -0.13 (-0.16, -0.10) |
| Low Lean Only | 1404 (9%) | -1.68 (-1.72, -1.65) | -1.57 (-1.63, -1.51) | -0.77 (-0.82, -0.73) |
| Obese Only | 6428 (43%) | 0.49 (0.46, 0.52) | -0.47 (-0.50, -0.44) | 1.12 (1.10, 1.14) |
| Sarco Obese | 543 (4%) | -1.68 (-1.73, -1.62) | -2.71 (-2.81, -2.62) | 0.49 (0.46, 0.51) |
| **Health ABC** |  |  |  |  |
| Normal | 1249 (44%) | -0.28 (0.63) | -0.11 (0.78) | -0.040 (0.54) |
| Low Lean Only | 192 (7%) | -1.66 (0.49) | -1.69 (0.70) | -0.58 (0.64) |
| Obese Only | 1317 (46%) | 0.017 (0.68) | -0.74 (0.79) | 0.84 (0.42) |
| Sarco Obese | 86 (3%) | -1.60 (0.46) | -2.54 (0.69) | 0.45 (0.39) |

Supplementary Table 3: Body composition T-Scores across body composition categories defined based on ALMI_FMI_ and FMI categorization method in NHANES and Health ABC.

|  | **N (%)** | **ALMI T** | **ALMI_FMI_ T** | **FMI T** |
| --- | --- | --- | --- | --- |
| **NHANES** | 14850 |  |  |  |
| Normal | 9025 (61%) | -0.31 (-0.33, -0.29) | -0.16 (-0.19, -0.13) | -0.11 (-0.13, -0.083) |
| Low Lean Only | 769 (5%) | -2.02 (-2.07, -1.97) | -2.69 (-2.74, -2.63) | -0.087 (-0.14, -0.030) |
| Obese Only | 4761 (32%) | 0.84 (0.81, 0.87) | -0.23 (-0.26, -0.19) | 1.32 (1.30, 1.33) |
| Sarco Obese | 293 (2%) | -0.99 (-1.07, -0.91) | -2.61 (-2.71, -2.52) | 1.21 (1.16, 1.26) |
| **Health ABC** | 2844 |  |  |  |
| Normal | 1882 (66%) | -0.40 (0.63) | -0.41 (0.82) | 0.054 (0.56) |
| Low Lean Only | 141 (5%) | -1.89 (0.52) | -2.54 (0.50) | -0.054 (0.64) |
| Obese Only | 766 (27%) | 0.38 (0.57) | -0.51 (0.76) | 1.07 (0.32) |
| Sarco Obese | 55 (2%) | -1.00 (0.50) | -2.54 (0.61) | 1.11 (0.31) |

Supplementary Table 4: Percent of participants from NHANES with difficulty completing daily life tasks across body composition categories. Analysis adjusted for sex and race only (T-Score analysis).

|  | **Climb 10 Steps** | **Stoop Down** | **Lift Objects** | **Stand from Chair** | **Stand for Long Periods** | **Walk a ¼ Mile** |
| --- | --- | --- | --- | --- | --- | --- |
| **Low ALMI & High % Fat** | |  |  |  |  |  |
| Normal | 13.6 | 42.4 | 22.1 | 17.6 | 36.0 | 20.1 |
| Low Lean Only | 20.3** | 44.1 | 30.3*** | 21.5* | 41.6** | 24.0 |
| Obese Only | 25.8*** | 60.0*** | 27.4** | 29.1** | 48.9*** | 32.1*** |
| Sarcopenic Obesity | 22.0** | 52.5** | 33.0** | 27.2*** | 47.4*** | 29.8** |
| **Low ALMI_FMI_ T-Score & High FMI** | |  |  |  |  |  |
| Normal | 14.2 | 42.4 | 23.2 | 17.4 | 36.1 | 20.0 |
| Low Lean Only | 24.3*** | 53.1*** | 33.5** | 28.9*** | 50.0*** | 29.3** |
| Obese Only | 30.0*** | 64.9*** | 29.0*** | 33.0*** | 53.1*** | 36.5*** |
| Sarcopenic Obesity | 28.9*** | 60.3*** | 29.9* | 29.0* | 50.0*** | 37.0*** |

Supplementary Table 5: Ordinal regression model evaluating risk of greater disability among different categories of body composition by different methods adjusting for age, sex, and race (T-Score analysis from NHANES).

|  | **Risk of Greater Reported Disability** |
| --- | --- |
| **ALMI & High % Fat** | OR (95% CI) |
| Normal | 1 (reference) |
| Low Lean Only | 1.15 (0.94, 1.40) |
| Obese Only | 1.72 (1.49, 1.98)*** |
| Sarcopenic Obesity | 1.51 (1.16, 1.96)** |
| **ALMI_FMI_ T-Score & FMI** |  |
| Normal | 1 (reference) |
| Low Lean Only | 1.68 (1.31, 2.17)*** |
| Obese Only | 2.21 (1.94, 2.53)*** |
| Sarcopenic Obesity | 1.88 (1.42, 2.48)*** |

Supplementary Table 6: Linear regression and Cox proportional hazards model evaluating risks of poor physical function and incident disability adjusted for age, sex, and race. Table shows predicted mean of the Health ABC physical function score from regression models in each group and HR for incident disability.

|  | **Health ABC Performance Score** | **Risk of Incident Disability**  N=2738, 2097 events |
| --- | --- | --- |
| **ALMI & % Fat** | Predicted Mean (95% CI) | HR (95% CI) |
| Normal | 2.30 (2.27, 2.33) | 1 (reference) |
| Low Lean Only | 2.21 (2.14, 2.28)* | 1.22 (0.98, 1.42) |
| Obese Only | 2.12 (2.10, 2.15)*** | 1.44 (1.32, 1.41)*** |
| Sarcopenic Obesity | 2.00 (1.90, 2.11)*** | 1.32 (1.03, 1.70)* |
| **ALMI_FMI_ T-Score & FMI** |  |  |
| Normal | 2.27 (2.25, 2.30) | 1 (reference) |
| Low Lean Only | 2.05 (1.97, 2.13)*** | 1.24 (1.02, 1.50)** |
| Obese Only | 2.08 (2.04, 2.11)*** | 1.54 (1.39, 1.69)*** |
| Sarcopenic Obesity | 1.92 (1.79, 2.05) *** | 1.49 (1.12, 2.00)** |
